# Investigation of Wall Shear Stress in Cardiovascular Research and in Clinical Practice—From Bench to Bedside

**DOI:** 10.3390/ijms22115635

**Published:** 2021-05-26

**Authors:** Katharina Urschel, Miyuki Tauchi, Stephan Achenbach, Barbara Dietel

**Affiliations:** Department of Medicine 2—Cardiology and Angiology, Friedrich-Alexander-University Erlangen-Nürnberg (FAU), Universitätsklinikum, 91054 Erlangen, Germany; Katharina.Urschel@uk-erlangen.de (K.U.); miyuki.tauchi@uk-erlangen.de (M.T.); Stephan.Achenbach@uk-erlangen.de (S.A.)

**Keywords:** atherosclerosis, wall shear stress, endothelial activation, plaque, rupture, mechanotransduction, glycocalyx

## Abstract

In the 1900s, researchers established animal models experimentally to induce atherosclerosis by feeding them with a cholesterol-rich diet. It is now accepted that high circulating cholesterol is one of the main causes of atherosclerosis; however, plaque localization cannot be explained solely by hyperlipidemia. A tremendous amount of studies has demonstrated that hemodynamic forces modify endothelial athero-susceptibility phenotypes. Endothelial cells possess mechanosensors on the apical surface to detect a blood stream-induced force on the vessel wall, known as “wall shear stress (WSS)”, and induce cellular and molecular responses. Investigations to elucidate the mechanisms of this process are on-going: on the one hand, hemodynamics in complex vessel systems have been described in detail, owing to the recent progress in imaging and computational techniques. On the other hand, investigations using unique in vitro chamber systems with various flow applications have enhanced the understanding of WSS-induced changes in endothelial cell function and the involvement of the glycocalyx, the apical surface layer of endothelial cells, in this process. In the clinical setting, attempts have been made to measure WSS and/or glycocalyx degradation non-invasively, for the purpose of their diagnostic utilization. An increasing body of evidence shows that WSS, as well as serum glycocalyx components, can serve as a predicting factor for atherosclerosis development and, most importantly, for the rupture of plaques in patients with high risk of coronary heart disease.

## 1. Introduction: Mechanical Forces in the Vascular System and Atherosclerosis

Several physiological functions, such as the regulation of homeostasis, vascular tone, and vascular integrity, are modulated by different mechanical forces acting on the arterial wall. Apart from their role in homeostasis, increasing evidence indicates the involvement of hemodynamics in vascular disease development. Transduction of mechanical forces into cellular responses is suggested to be crucial in the pathology of atherosclerosis, affecting both disease onset and progression. The main forces acting on the arterial wall include tensile stress induced by blood pressure and—most importantly in atherogenic hemodynamics—so-called wall shear stress (WSS), a tangential force to the vessel wall induced by blood flow [1].

**Figure 1:**
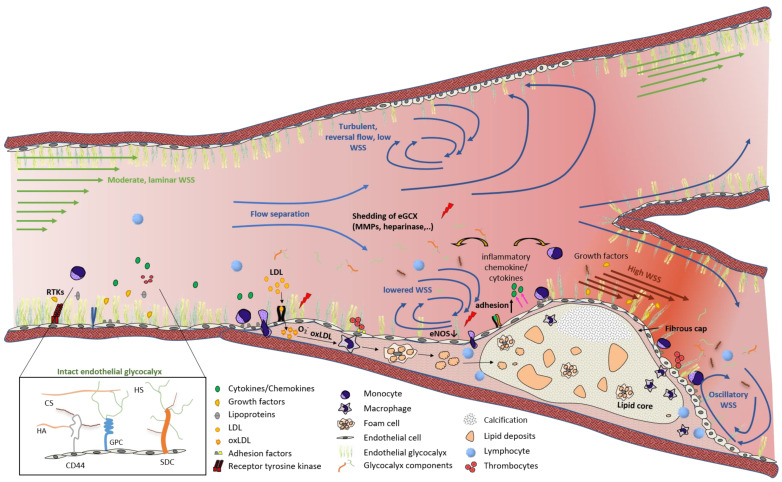
Flow and WSS patterns at arterial bifurcations, leading to endothelial dysfunction and atherosclerotic plaque development. In straight vessel segments, physiological WSS with laminar flow leads to a quiescent EC phenotype with an intact eGCX. At bifurcation sites, flow separation causes turbulent and reversal flow with lowered WSS at the outer vessel wall and the plaque shoulder regions, whereas high WSS occurs at the throat of the plaque. These different flow patterns and the resulting WSS gradient result in eGCX degradation and endothelial dysfunction, characterized by the induction of inflammatory signaling cascades, hence further promoting plaque progression. CS: chondroitin sulfate, GPC: glypican, HA: hyaluronic acid, HS: heparan sulfate, RTK: receptor tyrosine kinase, SDC: syndecan.

The magnitude of this frictional force mainly depends on blood viscosity and velocity gradients, as well as on vessel geometry, such as the luminal radius. The magnitude and direction of WSS are recognized by mechanosensors on the endothelium, the inner lining of the arterial wall, and are transduced as biochemical signaling. For example, induced mechanotransduction leads to an activation of endothelial NO synthase (eNOS) in endothelium, followed by an increase in the release of endothelial nitric oxide (NO), which in turn modulates cellular contractility and consequently vessel dilation. In addition to eNOS, the expression of numerous genes involved in cell morphology, adhesion, proliferation, and thrombosis is regulated by shear-stress-induced mechanotransduction, indicating the important role of WSS in both the physiological stage and during progression of vascular diseases. Accordingly, the involvement of WSS in atherosclerotic plaque development was already demonstrated decades ago in autopsy specimens of human carotid bifurcations, in which atherosclerotic lesions mainly occur in regions which are characterized by low WSS and flow separation (Figure 1). These athero-prone conditions can be found at the outer wall of arterial branches and bifurcations, i.e., the carotid sinus. The observed findings led to the hypothesis that mechanical fluid forces might be involved in atherosclerosis development.

To investigate the role of WSS in atherogenesis, experimental flow systems were established and combined with diverse imaging modalities to measure WSS in both in vivo and in vitro settings. Additionally, specified animal models have served to explore the impact of hemodynamics on disease progression. An enormous research effort has been made during recent decades, which has continuously increased our knowledge about the role of WSS in vascular diseases, including mechanosensing mechanisms, endothelial activation, immune modulation, and the initiation and development of atherosclerosis. With regard to clinical applications, WSS measurement has improved risk stratification in patients with carotid and coronary artery disease.

In this review, we briefly revisit the discovery of atherogenic hemodynamics, and describe the commonly used and current techniques to measure WSS, both in the clinical setting and in cardiovascular research, to investigate how hemodynamics are involved in atherogenesis and plaque progression. Paying particular attention to endothelial mechanoactivation, we will discuss the current understanding of its role in atherogenesis, highlighting the function of the endothelial glycocalyx (eGCX) as a mechanosensing structure.

## 2. Experimental Animal Models to Investigate the Role of Hemodynamics in Atherogenesis

### 2.1. Atherogenic Animal Models

As early as in the beginning of 20th century, researchers tried to experimentally induce atherosclerosis in animals. A well-known example is the Anitschkow rabbit model. Anitschkow fed rabbits with a high-fat diet and showed atherosclerosis-like lesions in the aorta in 1913 [2]. Later, experimentally-induced hyperlipidemia by means of a high-cholesterol diet or infusion of cholesterol was demonstrated reliably to cause atherosclerotic lesions in many different animal species.

The Watanabe-heritable hyperlipidemic (WHHL) rabbit model was established through the inbreeding of spontaneous hyperlipidemic rabbits [3]. WHHL rabbits carry a mutation in the low-density lipoprotein receptor (LDLr) which causes an 8–14-fold increase in blood cholesterol levels compared with wild-type rabbits [4]. In humans, hypercholesteremia had been considered a risk factor for atherosclerosis and cardiovascular events, which was confirmed by the Framingham study [5]. In this regard, WHHL rabbits effectively model a core mechanism of human atherogenesis as a consequence of lipid dysregulation. Indeed, human familial hypercholesterolemia is caused by defective LDLr [6].

After targeted gene knock-out technology became available, many mouse models were generated, including apolipoprotein E (ApoE) and LDLr deletion, both of which are widely used in atherosclerosis research to date (reviewed in [7]). A tremendous amount of both in vitro and in vivo studies supports the hypothesis that high circulating cholesterol is one of the main causal factors for atherosclerosis.

### 2.2. Atherogenic Hemodynamics

The establishment of animal models with genetically altered lipid metabolism has robustly fostered progress in atherosclerosis research. Although these models shed light on the involvement of hyperlipidemia in the pathogenesis of atherosclerosis, this cannot explain the localization of plaques in vessels. It has been shown that atherosclerotic lesions are localized in vessel segments proximal to bifurcations and arterial branches in rabbits [8], swine [9], ApoE-deficient mice [10], and in humans [11]. Furthermore, it was documented that local hemodynamic forces on the arterial wall affect the morphologic phenotype of the endothelium. Endothelial cells (ECs) in casted rabbit aortas showed differing morphology depending on the respective vessel segment [12], which is assumed to arise from different local WSS patterns. Baker et al. surgically modified blood flow in the rabbit aorta and confirmed that WSS changed the morphology of arterial ECs in vivo [13]. Such phenotypic changes were also confirmed by in vitro studies, which demonstrated that cultured ECs are in a cobblestone-like structure under static conditions, whereas they are in an aligned, spindle structure under uniform, laminar flow conditions [14].

Based on these observations, it was hypothesized that mechanical fluid forces modify the endothelial phenotype and are involved in atherosclerosis development. To address this issue, promising in vivo and in vitro models were established to investigate the impact of different hemodynamic conditions on vascular cells and cellular interactions, which further drove atherosclerosis research.

### 2.3. Characterization of Hemodynamics

The utilization of in vitro culture models to study the role of hemodynamics in atherogenesis requires a solid understanding of the flow conditions in vivo. Great effort has been made to characterize hemodynamic conditions in the vascular system in vivo, enabling researchers to investigate the regulation of EC functions by hemodynamics in vitro in relation to atherogenesis.

In experimental animal models, flow velocity has been investigated both invasively and non-invasively in vivo or also ex vivo. Altobelli and Nerem perfused hearts of baboons and dogs ex vivo and measured fluid velocity in epicardial coronary arteries using a pulsed ultrasonic Doppler velocimeter [15]. They showed that flow velocity was determined by the vessel geometry and that vessel segments distal to bifurcations are characterized by disturbed flow. Furthermore, using postmortem human carotid arteries, plaque localization was shown to correlate with low flow velocity [16]. However, the experimental manipulation of flow velocity in vivo did not affect lipid deposition in the aorta, possibly because vessels adapted to velocity changes through modulation of the lumen diameter [17]. Indeed, flow velocity is not the only factor affecting EC morphology and function. These other factors include oscillating flow patterns; the turbulence of the flow direction; tensile stress; and shear stress, which is determined (amongst others) by flow velocity, to name a few. Computational fluid dynamics (CFD) modeling represents one of the attempts to simulate these complex hemodynamic conditions to investigate the impact of flow dynamics on disease progression, such as atherosclerosis.

Using a reconstructed vessel geometry, CFD modelling mathematically calculates and models blood flow, using the data obtained with angiography and other imaging modalities. These imaging techniques have been widely used in humans for diagnosis and research purposes. These techniques have been challenging in experimental animal models so far, but recent progress in the development of preclinical imaging approaches has expanded their application [18]. Riemer et al. used contrast-enhanced ultrasound image velocimetry in rabbits and demonstrated the feasibility of spatiotemporal WSS mapping [19]. Riedl et al. computed WSS from 17.6 Tesla MRI data that were longitudinally collected at different sites of the aorta from ApoE knockout mice fed with a Western-type diet, demonstrating that low WSS was associated with an increased local plaque burden [20]. Suo et al. used micro-computer tomography (CT) to describe the geometry of the mouse aorta, as well as pulsed-Doppler ultrasound to measure flow velocity, and computationally determined its WSS [21]. In that study, the vessels from different areas of the aorta were sampled and higher expression of vascular cell adhesion molecule (VCAM)-1 and intercellular adhesion molecule (ICAM)-1, examples of markers for endothelial activation, were observed at areas exposed to low WSS.

### 2.4. Atherogenic Animal Models: Beyond Lipid

Using atherogenic animal models, the pathophysiology of atherogenesis in terms of lipid disturbance has been intensively investigated. In vitro flow models, alone or combined with CFD models, have contributed to our understanding of endothelial dysfunction and immune modulation by hemodynamics. However, a limited number of in vivo models are available to address the question of how flow dynamics affect molecular and cellular functions of ECs, which ultimately lead to endothelial dysfunction and atherosclerosis development. To date, knowledge is limited as to which flow-induced or flow-disturbed functions alter lipid metabolism, transport, and biosynthesis. Nonetheless, changes in lipid profile have been shown to affect hemodynamics: ApoE-knockout mice demonstrate an elevated pulse wave velocity and mitral and aortic flow velocity in comparison to wild-type mice, even though heart rate and blood pressure were shown to be unaltered [22], suggesting an association between lipid disturbance and WSS. It is not clear which is the main factor to influence these hemodynamics changes, whether it is a direct effect of altered lipid metabolisms or an indirect effect, e.g., caused by changes in vessel geometry due to emerging plaques.

Flow modulation in animal models has often been induced surgically. A common method is a partial ligation of the carotid artery. Some attempts have been made to modulate blood flow surgically to investigate its consequence in terms of atherogenesis. Perturbation of flow can be induced by placing a constrictive cuff around the common carotid artery. In this experimental setting in mice, electrical injury was first induced at the intima of the common carotid artery to mimic plaque rapture and thrombosis; then, oscillating WSS was induced by a cuff. The recruitment of neutrophils to the injured site was significantly increased with the constrictive cuff, indicating that turbulent flow enhanced an athero-susceptible phenotype of the endothelium [23]. Another method is a partial unilateral ligation of the common carotid artery, which can induce ectopic oscillating shear stress at the ligated artery [24]. Wang et al. used this surgical modulation of flow in ApoE-deficient mice and demonstrated that the disturbed flow induced proatherogenic mechanotransduction, which was associated with pronounced atherosclerotic lesion formation [25]. Some studies used this ligation model and showed fragmentation and thinning of elastic fibers and vascular stiffness at sites of disturbed flow, which can be evaluated by ultrasound elastography and may possibly be used to assess plaque vulnerability [26].

In humans, plaque formation occurs spontaneously at an early age and progresses slowly with normal circulating lipid levels [27]. Therefore, hyperlipidemic animal models with atherosclerosis induced by a high-fat diet or specific gene mutations may not necessarily model well the pathology of human atherosclerosis. Interestingly, there are some animal models in which atherosclerosis can develop spontaneously, or which are susceptible to plaque formation under a high-fat diet, even though the genetic predispositions of these animals are not toward disturbed lipid metabolism. For example, spontaneous fatty streaks are formed in swine in early life and gradually develop with aging. The whole process can be accelerated by a cholesterol-rich diet [9]. These animals better model the actual course of disease progression in human atherosclerosis.

Another animal model, in which atherosclerosis develops spontaneously and independently from hyperlipidemia, is White Carneau (WC) pigeons. WC pigeons are a well-characterized avian model of atherosclerosis. They present spontaneous and inherited atherosclerosis development, although their blood lipid profile does not apparently differ from that of Show Racer (SR) pigeons, which are atherosclerosis-resistant. In WC pigeons, differences compared with those in SR pigeons include increased glycosaminoglycans, especially chondroitin-6-sulfate (reviewed in [28]).

An increasing number of studies has demonstrated that glycosaminoglycans on the surface of ECs are atheroprotective. The eGCX is the extracellular layer of ECs, consisting of glycosaminoglycans, proteoglycans, and glycoproteins. It is well documented that hemodynamics affect the GCX in vitro (see Section 3). The functional significance of this has been confirmed in several in vivo studies. Harding et al. showed, in a partial ligation model in mice, an increased degradation of the eGCX at sites of disturbed flow, which was associated with enhanced endothelial activation, indicated by decreased caveolin-1 and eNOS levels [29]. As an intact eGCX structure is assumed to have atheroprotective effects, its enhanced degradation might be involved in proatherogenic mechanotransduction under oscillatory WSS. In Table 1, characteristics of oscillatory WSS are compared with those of low and high WSS.

Apart from an impaired eGCX, oscillatory WSS, which is characterized by bidirectional blood flow containing regions with flow separation, recirculation, and even flow stagnation, exert an activated, proinflammatory phenotype in ECs. Mechanoactivation of transcription factors driving cell proliferation and inflammation causes endothelial dysfunction and intimal thickening, which are the main steps in the onset of atherosclerosis. In contrast, ECs exposed to both moderate or high WSS have an intact eGCX and are characterized by reduced permeability compared with oscillatory WSS. As components of the eGCX play a key role in mechanosensing, its degradation might be involved in the induction of proatherogenic mechanotransduction under disturbed flow conditions.

To investigate the role of the GCX, there are some genetically modified mouse models for certain GCX molecules. Using these models, syndecan-1 and -4 were shown to be atheroprotective [30,31]. Furthermore, biglycan was described to stabilize plaques, protecting the lesions against rupture [32].

## 3. In Vitro Systems to Model Flow Dynamics and Endothelial Wall Shear Stress (WSS)

Apart from animal models, in vitro studies are extensively used to investigate in vivo flow dynamics. The accumulated understanding of hemodynamics from in vivo studies has been successfully utilized to model atherogenic flow in vitro. Various and creative in vitro cell culture models have been developed, which continuously contribute to increasing our knowledge of molecular and cellular aspects of atherogenesis.

In vitro flow-through culture systems serve as a model to investigate cellular responses to flow-induced mechanostimulation. By mimicking the hemodynamics of the vascular system, mechanotransduction and its role in atherogenesis can be explored with regard to diverse aspects, such as cellular interactions to address cell adhesion and migration or functional analyses, e.g., cell permeability or contractility. As numerous factors, such as vessel geometry, blood velocity and viscosity, blood pressure, and arterial stiffness, affect hemodynamics in the vasculature, in vitro systems are not able to mimic all aspects of such a complex environment. However, these models offer new perspectives to address specific issues, e.g., the investigation of cellular interactions under different patterns of shear stress. 

In vitro systems bear the advantages of being well standardized, easy to handle, in many cases less expensive, and ethically tenable. Additionally, such systems enable the manipulation of single parameters to focus on particular aspects, without interfering with the diverse variables existing in a complex in vivo setting. The established in vitro systems can be applied in combination with CFD to calculate hemodynamic parameters and to investigate force-induced cellular responses to flow [33,34]. With the knowledge obtained from in vitro as well as in vivo studies, Dabagh et al. [35] computationally modeled EC mechanics to investigate the impact of flow direction on cellular force transmission. Incorporating diverse cell characteristics, such as form, cytoskeletal organization, eGCX, and adherence junctions, the applied model demonstrated the effect of atherogenic oscillatory shear stress on subcellular structures of EC, which underlie atherogenic mechanotransduction.

In vitro models can be classified in several ways. In this review, we will describe the most commonly used two-dimensional (2D) and three-dimensional (3D) models and their assembly, considering the advantages and disadvantages of each method. As the endothelium plays a key role in transducing shear stress into biochemical signaling, we focus on models used to investigate endothelial mechanotransduction of WSS, paying particular attention to the eGCX, an essential surface structure for mechanosensing.

### 3.1. Two-Dimensional (2D) In Vitro Models

In the 1970s and 1980s, parallel plate flow chambers (PPFCs), as well as cone and plate viscometers, were first fabricated for the application of flow on a cellular monolayer to investigate various responses to mechanostimulation by flow, such as histidine decarboxylase production [36] and leukocyte adhesion [37]. During recent decades, major efforts have been made for an adjustment of these systems to get closer to the existing physiological conditions of the vascular system. Established setups enabled the variation of diverse parameters to control, e.g., surface composition and geometry of the flow chamber, cell types, and the applied fluid dynamical conditions. PPFCs consist of a cell culture surface that can be coated with different matrices and a silicon gasket and a cover. The device requires an inflow and outflow connection to a pump system. The cone-and-plate-viscometer uses similar settings: this setup uses a rotating cone or cylinder for shear stress generation instead of a pump system.

A broad range of in vitro systems has been further developed to investigate hemodynamics-induced mechanotransduction during recent decades, e.g., parallel-plate chambers [38,39]. As cellular functions often depend on cellular shape and orientation, which are determined by flow-induced hemodynamics, effort has been made to model complex blood flow observed in in vivo settings. Atheroprone oscillatory flow can be induced using the cone and plate shear devices [40] or parallel plate flow chambers [41]. Sinha et al. [42] have developed a medium-throughput cell culture device that can produce variously oriented anisotropic biaxial strains by stretching a cell-growing surface membrane, which models strains caused by vascular wall changes in shape and mechanical properties, as seen in various vascular diseases, such as atherosclerosis. The device demonstrated that such strains in various conditions resulted in different alignment responses of ECs. These findings underline that mechanotransduction depends on a complex interplay of diverse forces. Meza et al. [43] also analyzed the effects of pulsatile shear stress and cyclic tensile strain on ECs and found that both forces, but not synergistically, increased effects on EC morphology and activation, indicated by the phosphorylation of platelet endothelial cell adhesion molecule (PECAM)-1 and increased surface expression of ICAM-1.

The identification of mechanosensitive structures on the apical side of cells is of particular interest, because cellular sensing of shear stress initiates an intracellular signaling cascade to transform the mechanical stimulus into a biochemical response. Using a bifurcation flow-through model, the effects of different flow conditions and shear stress patterns, as well as of a WSS gradient, can be explored in one device, for example, the flow-induced modulation of diverse atherogenic mechanosensing molecules on the endothelial surface, such as selectins, ICAM-1, vascular growth factor receptor (VEGFR)-2 [44,45], or their functional significance in leukocyte adhesion [46].

**Figure 2:**
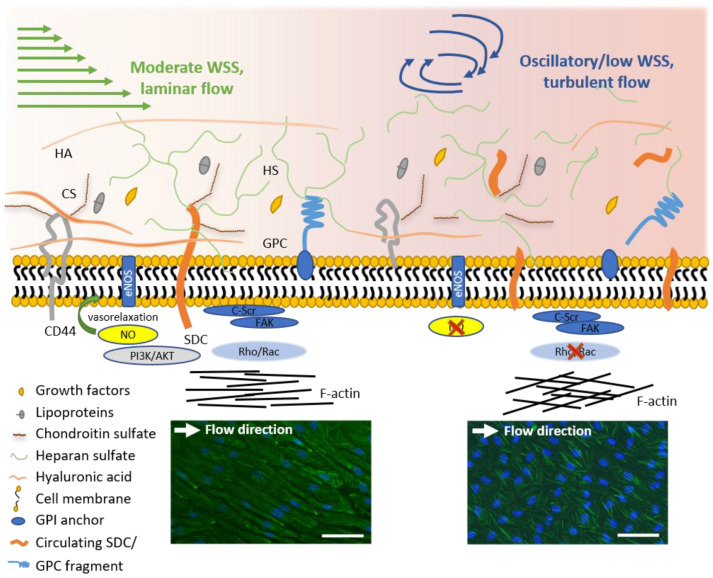
GCX signaling under different WSS conditions. Shedding of the eGCX occurs under low or oscillatory WSS with turbulent flow, thereby affecting different intracellular signaling pathways, e.g., NO-synthase and cytoskeleton arrangement. Microphotographs of immunofluorescence staining show human umbilical vein ECs after 24 h of laminar (left) or non-uniform, turbulent (right) flow. Nuclei are stained in blue (DAPI) and F-actin is stained in green (phalloidin Alexa Fluor 488). Scale bar: 100 µm; AKT: protein kinase B, CS: chondroitin sulfate, eNOS: endothelial nitric oxide synthase, GPC: glypican, HA: hyaluronic acid, HS: heparan sulfate, NO: nitric oxide, c-Scr: proto-oncogene tyrosine-protein kinase Src, FAK: focal adhesion kinase, PI3K: phosphoinositide-3phosphate, Rac: Ras-related C3 botulinum toxin substrate, RhoA: Ras homolog family member A, SDC: syndecan.

During the last years, mechanosensing by means of a cellular surface structure, the eGCX, gained increasing attention (reviewed in [47,48]). For example, in a recent study using PPFC, it was shown that endothelial expression of GCX components depends on hemodynamic conditions and in turn modulates the endothelial redox state in response to WSS [49]. Bartosch et al. observed NO production from rat fat pad ECs by physically applying mechanical force to individual components of eGCX [50]. They found that mechanical force on glypican-1 and heparan sulfate caused significantly increased NO production, concluding that these two eGCX components transduce mechanical signals into intracellular NO-signaling. The impact of different WSS conditions on the eGCX and the resulting intracellular signaling pathways which affect NO production or cytoskeleton reorganization are illustrated in Figure 2. The above described flow chamber systems can be combined with diverse imaging approaches, such as laser Doppler velocimetry or live cell microscopy, to characterize the local WSS pattern and the cellular response to the applied mechanical force. The establishment of atomic force microscopy (AFM) enabled researchers to investigate shear stress-induced modulation of the cell surface and morphology on a single-cell level [51].

The influence of different hemodynamic properties can also be analyzed in real-time by means of impedance measuring in cells, which is frequency-dependent and was further developed into the Electric Cell-Substrate Impedance Sensing (ECIS) system. It can be used to measure a variety of cellular properties, e.g., behavior under flow [52]. Another setup is the combination of a small impedance chip with an orbital shaker to produce oscillatory shear, which allows the measurement of trans-endothelial resistance against varying shear stress conditions [53]. Furthermore, microfluidic chips, also known as “organ-on-a-chip” technology, is a further advanced form of in vitro systems, which may contribute to our understanding of EC responses to hemodynamics [54] (reviewed in [55,56]).

All the obtained adaptions of the principal experimental setups follow the same goal—for in vitro models to mimic the physiological conditions in vivo as closely as possible. To achieve this goal, more differentiated and more sensitive molecular tools have been developed, either on a single-cell level or in the form of complex structures composed of a variety of cells and matrices to construct “vascular beds”.

### 3.2. Three-Dimensional (3D) In Vitro Models

To analyze cellular functions under consideration of the respective vessel geometry, 3D models have been utilized. In these models, matrix gels provide a 3D scaffold for cells to grow and form a structure. ECs seeded and cultured on a matrix composed of fibrin or collagen can invade the matrix and form tubular structures, which is used as a model for angiogenesis. Bayless et al. used this model and demonstrated that endothelial sphingosine-1-phosphate (S1P) induced matrix metalloproteinases (MMPs) to promote angiogenesis [57]. The 3D culture system enables the co-culture of several cell types with ECs. Furthermore, this system can be combined with flow and has been used in a wide variety of contexts to date, e.g., for the characterization of intercellular signaling during pre-vascular network formation [58] and to mimic pressure-volume changes seen in the left ventricle in encapsulated cardiac cells [59]. Apart from 3D scaffold gels described above, there have been attempts to provide a tubular, vessel-like scaffold structure to ECs. In 1995, Ott et al. [60] used a 1.5-mm polyurethane vascular graft as a scaffold and seeded it with bovine aortic endothelial cells. The graft was fixed in a flow chamber and shear stress was applied. They analyzed the effect of different shear rates for up to 6 days on the endothelial monolayer and compared the clotting time of whole blood on ECs with or without shear stress exposure. In the vascular grafts with shear stress exposure, a significantly prolonged clotting time was observed compared with those without shear stress. Farcas et al. [61] sought to fabricate a simplified, yet anatomically realistic, tubular in vitro model of the human right coronary artery with a Sylgard^TM^ 184 silicone elastomer, casted from a postmortem human heart. Steady flow was applied on human abdominal aortic (HAA) ECs seeded in the model to determine its effect on cell morphology at different time points, demonstrating the association of shear forces with cell elongation in the direction of the applied flow.

3D printing and tissue engineering are promising approaches to fabricating new 3D tubular devises for the in vitro modeling of hemodynamics. 3D microfluidic chambers [62,63], as well as synthetic tubular branching vessels with complex geometries, namely phantoms [64], have been fabricated and are intended to be employed in cardiovascular research. Furthermore, artificially stenosed vessels or valves in a microfluidic system have been established, often combined with CFD, focusing on hemodynamic properties depending on vessel diameter and/or the degree of stenosis [65,66], which allows for the investigation of the effect of different degrees of stenosis on WSS or shear stress gradients in atherosclerotic lesions.

To understand the effect of arterial stiffness on ECs, various polyacrylamide protein gels have been tested, showing that decreased stiffness in matrices, mimicking young and healthy vessels, can induce an antiatherogenic phenotype in ECs under flow conditions [67,68]. The implicated signaling pathway seems to involve Ras homolog family member (Rho)A kinase, eNOS, and extracellular signal-regulated kinases (ERK)1/2 [67]. As one of the possible mechanosensors responding to WSS in ECs seeded on these soft substrates, Galie et al. demonstrated the eGCX component, hyaluronic acid, plays a role [68]. Cooper et al. [69] used a 50% stenosis model to investigate the effect of WSS on eGCX degradation by MMPs. Furthermore, to test GCX modification in vitro, the 3D Sylgard^TM^ 184 culture model was also utilized [70].

### 3.3. Translational Application of the In Vitro Models

Among the above-described in vitro models, the artificially constructed 3D models in particular may represent a bridge to their translational application, because they can closely simulate physiological conditions or patient-specific geometry (reviewed in [71,72]). To date, hemodynamics research has most frequently been carried out and has elucidated atherogenic hemodynamics ex vivo. Although vessels from animals have been used in most ex vivo models, there is an increasing number of studies which have successfully utilized human vessels, such as the human saphenous vein [73,74,75] or human umbilical vein [76,77], to overcome interspecies differences.

## 4. Measurement of Wall Shear Stress (WSS) in Clinical Settings

Although both in vitro models and in vivo experimental animal studies have focused on investigating the role of hemodynamics in atherogenesis, measurements of WSS in clinical practice is of particular interest for risk stratification in coronary and carotid atherosclerosis. The former understanding that the degree of vessel stenosis has the greatest effect on the risk of future ischemic events is now considered obsolete, and it is now widely accepted that local hemodynamics play a more crucial role in risk prediction. Various techniques have been developed to estimate WSS in patients. The frequently used techniques to measure WSS in coronary arteries shall be described in the following sections of this review, paying particular attention to their application, potential limitations in clinical practice, and important findings of respective studies.

### 4.1. Fusion of Intravascular Ultrasound (IVUS) with Angiography

Intravascular ultrasound (IVUS) was the first imaging approach established to measure WSS in coronary arteries of patients. Accordingly, in 1997, Krams et al. described the 3D reconstruction of coronary arteries through the fusion of angiography and IVUS, which was used to calculate WSS by means of CFD [78]. The conversion of reflected ultrasound waves into electrical signals enables the visualization of the arterial lumen and wall. Although IVUS does not permit the characterization of different plaque components, it offers a high penetration depth into the arterial wall [79]. Moreover, the development of virtual histology IVUS (VH-IVUS), which translates radiofrequency backscattered signal into tissue color images, compensates for some of the drawbacks of IVUS. Accordingly, plaque vulnerability can be estimated by distinguishing between different tissue types, such as fibrofatty or necrotic tissue [80].

**Figure 3:**
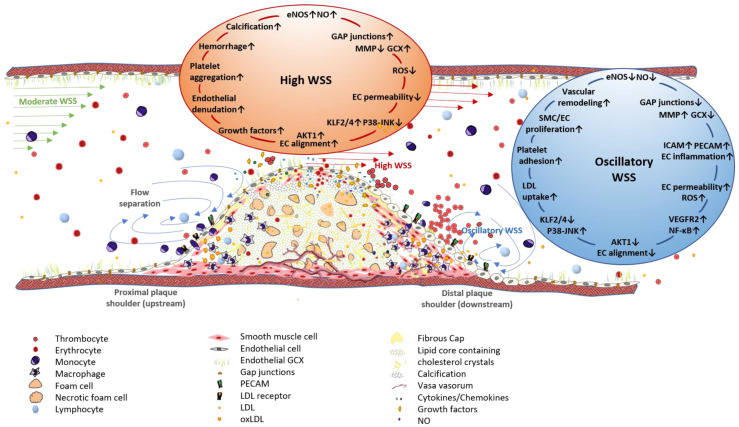
Association of WSS patterns of the arterial wall with plaque progression and vulnerability. Vessel stenosis caused by an advanced atherosclerotic lesion changes local WSS patterns. Moderate WSS, to which straight vessel segments are exposed, changes to high WSS at the throat of an arterial obstruction, whereas the downstream plaque shoulder is exposed to oscillatory WSS, characterized by flow separation and bidirectional flow. The endothelium, affected by high WSS, exhibits enhanced NO production due to mechanoactivation of eNOS, which is induced by the antiinflammatory transcription factor KLF2. Additionally, ECs show an axial alignment in the direction of blood flow, an almost intact GLX, and the low expression levels of adhesion molecules, such as PECAM and ICAM-1. Apart from its potentially atheroprotective effects, high WSS can promote plaque vulnerability by inducing platelet aggregation and endothelial denudation. Furthermore, plaques in vessel segments exposed to high WSS are characterized by enhanced intimal calcification. Oscillatory WSS is involved in plaque initiation and progression through the induction of EC and SMC proliferation and migration. Mechanoactivation of NF-ᴋB results in the increased expression of diverse proinflammatory genes, i.e., adhesion molecules and receptors for LDL uptake. EC alignment is inhibited and cells show a cobblestone-like morphology and increased permeability. Enhanced eGCX shedding due to increased MMP or sheddase levels facilitates the infiltration of immune cells from peripheral blood into the vessel wall. EC: endothelial cell, eNOS: endothelial nitric oxide synthase, GCX: glycocalyx, ICAM-1: intercellular adhesion molecule 1, KLF2: Krüppel-like factor 2, LDL: low density lipoprotein, NO: nitic oxide, PECAM: platelet endothelial cell adhesion molecule, SMC: smooth muscle cell, WSS: wall shear stress, ↑: upregulation, ↓: downregulation.

Using IVUS combined with angiography and blood flow measurements, Stone et al. demonstrated the association of low WSS with accelerated plaque progression and remodeling in the coronary arteries of patients with coronary artery disease (CAD) [81]. A carotid ligation model in ApoE^-/-^ mice showed that disturbed flow, which contains low and oscillatory shear stress, induced endothelial dysfunction and enhanced smooth muscle cell proliferation, whereas gene expression of eNOS and of antiinflammatory transcription factors such as Krüppel-like factor 2 (KLF2) was downregulated in those regions of the arterial wall [24]. Initial plaque development and progression at sites exposed to low WSS might be attributed to these effects. As shown in Figure 3, the endothelium exerts an activated, proinflammatory phenotype under low or oscillatory WSS, caused by mechanoactivation of the NF-ᴋB signaling pathway.

Although ECs show an axial alignment under physiological or high WSS, EC morphology changes under the disturbed flow conditions of oscillatory WSS. Cell alignment is lost and ECs have a round, cobblestone like shape (Table 1). Additionally, EC changes from a quiescent to an activated, proatherogenic phenotype, characterized by an impaired eGCX due to shedding (Figure 2) and increased permeability and proliferation.

Apart from its role in plaque initiation and progression, oscillatory WSS is also assumed to promote plaque vulnerability. In the Providing Regional Observations to Study Predictors of Events in the Coronary Tree (PROSPECT) trial, WSS evaluation using radiofrequency IVUS verified the association of low WSS with future non-culprit major adverse cardiovascular events (NC-MACE) in patients with acute coronary syndrome (ACS), emphasizing the prognostic value of WSS [82]. An increased risk of plaque rupture might arise from the pronounced proliferative response of ECs under oscillatory WSS, resulting in neovascularization, which promotes plaque rupture.

High WSS, which the most stenotic site of arterial obstructions is exposed to (Figure 3), has also been shown to be involved in plaque destabilization [80,83]: Analysis of plaque components and WSS using VH-IVUS and optical coherence tomography (OCT) showed that ruptured plaques were exposed to higher WSS, especially at segments proximal to the constriction site [80]. Increased shear forces at these regions are estimated to promote plaque vulnerability through the induction of endothelial denudation, vascular inflammation, fibrous cap (FC) thinning, and thrombus formation due to the attachment of platelets to the exposed vascular tissue (Figure 3). Indeed, thrombi have frequently been found between the throat and the distal plaque shoulder, where high WSS with laminar flow turns into oscillatory WSS with disturbed flow patterns.

### 4.2. Fusion of Optical Coherence Tomography (OCT) with Angiography

Apart from IVUS, OCT is a standardized imaging technique for 3D vessel reconstruction [84], which enables the tracing of the arterial lumen. As described for IVUS, OCT imaging also needs to be fused with coronary angiography to reconstruct 3D vessel geometry, followed by CFD-based WSS evaluation [85]. Due to its high resolution, OCT provides additional information about plaque vulnerability by visualizing, e.g., plaque neovascularization, thrombi, macrophage infiltration, and FC thickness [86]. In patients with ACS, the combination of OCT and angiography demonstrated enhanced plaque progression at regions of the coronary arteries exposed to low WSS, in agreement with former studies. Atherosclerotic lesions at these vessel segments were characterized by an increased lipid burden and a thin FC [86].

In OCT studies of CAD patients, high WSS has been shown to be associated with the presence of thin-cap fibroatheromas [87,88] and enhanced platelet activation at these sites [89], confirming its association with increased plaque vulnerability. Although thrombus initiation occurs at sites exposed to high WSS, the thrombus extends in segments distal to the high oscillating shear index (OSI) and low WSS (Figure 3), which is typically found at the distal shoulder region of the plaque [89].

In addition to risk stratification studies, the measurement of WSS also serves to unravel mechanisms involved in atherogenesis. The detection of cellular components involved in shear-stress-induced endothelial mechanotransduction has gained much attention during recent decades. By combining coronary angiography with OCT to evaluate the plaque lipid content and FC thickness in CAD patients, Nemoto et al. was able to show an association of plaque vulnerability with the serum concentration of GCX components [90]. In patients with ACS, analyses of serum peptidoglycan levels showed higher serum syndecan-1 levels in comparison to patients suffering from non-coronary chest pain and to a healthy control group [91]. As increased serum levels of this peptidoglycan have been described to result from enhanced eGCX degradation, the association of plaque progression with impaired eGCX can be assumed.

### 4.3. Three-Dimensional Quantitative Coronary Angiography (3D QCA)

In addition to 3D vessel reconstruction through the fusion of angiography with IVUS/OCT, 3D quantitative coronary angiography offers a simplified approach for CFD-based measurement of WSS. To reconstruct the arterial lumen, images of at least two X-rays of the respective vessel segment, which enable a 2D projection, are combined. As it is less time-consuming, 3D QCA can be performed during percutaneous coronary intervention (PCI). WSS measurement using 3D QCA in the coronary arteries of patients with stable CAD served to predict the risk of future cardiovascular events, such as myocardial infarction or cardiac death [92,93]. Apart from the use of fractional flow reserve as a prognostic factor, higher WSS in proximal segments of the occlusion site was shown to be predictive of future myocardial infarction. However, comparison between sole 3D QCA and the angiography–OCT fusion showed an overestimation of WSS by 3D QCA, which was caused by an undervalued luminal size [94]. Although it is less complicated and time-consuming, 3D QCA has not been established to date as a standard technique for WSS measurement due to its limited precision with regard to vessel reconstruction compared to the fusion models.

### 4.4. Coronary Computed Tomography Angiography (CCTA)

Moreover, noninvasive techniques have been established to identify atherosclerotic lesions prone to rupture due to local hemodynamic conditions. Coronary computed tomography angiography (CCTA) has been successfully applied as a noninvasive imaging approach for 3D vessel reconstruction to measure WSS in coronary arteries by using it in combination with CFD [95,96]. Compared to the established intravascular imaging techniques, the low spatial resolution is a main drawback of CCTA, hindering the visualization of small-diameter branches, which need to be included for the precise calculation of WSS [97]. Furthermore, it does not enable the characterization of plaque components, as is offered by IVUS radiofrequency or OCT. However, WSS measurement following CCTA-based vessel reconstruction can be easily accepted by patients for risk stratification compared with invasive imaging modalities [98]. Moreover, noninvasive imaging approaches to measure fluid shear stress can serve not only for risk stratification analyses, but also to explore shear stress mediated mechanosignaling in vivo during atherosclerosis development.

### 4.5. Near-Infrared Spectroscopy (NIRS) Intravascular Ultrasound

The development of near-infrared spectroscopy (NIRS) intravascular ultrasound enabled the additional characterization of plaque components, such as the lipid content. In a prospective cohort study, Waksman et al. identified CAD patients at high risk for NC-MACE by assessing non-culprit lesions, detected by NIRS intravascular ultrasound [99]. However, due to its limited penetration depth, this technique does not enable the visualization of deeper artery segments.

## 5. The Endothelial Glycocalyx (eGCX) and Its Role in Atherosclerosis as a Mechanosensor of WSS

The eGCX plays a key function in transducing mechanical forces into biochemical signaling. Although its role in shear stress mechanotransduction has been extensively explored in in vitro and experimental animal studies, investigations in humans are still rare, as the in vivo measurement of eGCX degradation remains challenging. The quantification of the perfused boundary region (PBR) of the eGCX, which is defined by the penetration depth of circulating blood cells inside the endothelial surface structure, by means of dark field capillaroscopy offers a technique to investigate GCX functions in vivo. Due to the barrier function of the intact eGCX, erythrocytes are kept at a certain distance from the endothelial membrane. Thus, eGCX thickness inversely correlates with microvascular perfusion of this boundary region [100]. Increased PBR values were described in patients with CAD and cerebral atherosclerosis, indicating an impaired eGCX during atherosclerosis progression [101,102].

Shedding and degradation of the eGCX can be induced by reactive oxygen species and proinflammatory cytokines, such as TNF-α and IL-1β, which activate metalloproteinases and other sheddases. Fragments of the shedded GCX enter into the blood stream, resulting in increased plasma levels of these components. Hence, the measurement of the plasma syndecan-1 level has been established as a circulating biomarker reflecting the rate of GCX shedding [103,104]. An impaired eGCX facilitates lipid accumulation and immune cell infiltration into the arterial wall, demonstrating the athero-protective impact of an intact GCX structure, which might serve as potential target in atherosclerosis therapy (reviewed in [47]).

To date, most research efforts have concentrated on in vitro and experimental animal studies, whereas only a few studies have investigated strategies for gylcocalyx protection in the clinical setting, although this number is increasing. In an isolated perfused heart model, Jacob et al. showed that degradation of the eGCX can be inhibited by albumin [105]. This colloid provides the endothelium with S1P, which inhibits GCX damage by suppressing MMPs. Tansfusion of fresh frozen plasma has also been described to protect the eGCX from degradation. Accordingly, its protective effect was demonstrated in an animal model of hemorrhagic shock or in patients with critical illness (reviewed in [106]). Both the protection of GCX degradation and the restoration of the damaged eGCX might be promising therapeutic strategies in the prevention and treatment of atherosclerosis.

In conclusion, the clinical measurement of WSS helps to identify patients at high risk of plaque progression and even of future plaque rupture. Clinical management of both early atherosclerosis and the advanced stage, bearing the increased risk of future ischemic events, might profit from the broader use of the established techniques to assess WSS.

## 6. Conclusions

Here, we reviewed previous studies on how the association between hemodynamics and atherosclerosis have been elucidated experimentally in vivo and in vitro. This review further examined the work in current and prospective clinical applications of the knowledge derived from these experiments, especially focusing on WSS and the eGCX as a key player in mechanotransduction. Apart from its clinical applications, the investigation of WSS and its mediated cellular responses also allows researchers to elucidate the role of diverse hemodynamic parameters and cellular components, such as the eGCX, in atherogenesis. Although the understanding of how mechanosensing and transduction is involved in atherosclerotic plaque development is still limited, several pathways induced by WSS have been identified using experimental animal models or in vitro models to mimic shear stress conditions in human vasculature. A “dynamic flow“ of knowledge about hemodynamics and the mediated mechanotransduction pathways contributes to a deeper understanding of vascular disease pathology and may be useful for the development of therapeutic strategies to prevent disease onset and progression.

## Figures and Tables

**Table 1:** Characterization of shear stress patterns in the human vasculature.

	High WSS	Low WSS	Oscillatory WSS
Vasoreactivity	Vasodilation	Vasoconstriction	Vasoconstriction
Flow direction/ Flow patterns	Unidirectional	Unidirectional	Bidirectional Flow separation, recirculation, stagnation Forward–reverse flow
Turbulence (Re)	>3500	<2300	>3000
Typical regions	Most narrow site of arterial obstructions Collateral arteries	Outer curve of bifurcations	Distal plaque shoulder
EC alignment in direction of flow	+	−	−
EC morphology	Elongated shape	Cobble-stone like shape	Cobble-stone like shape
eGCX shedding	−	+	+
Mechanoactivation of transcription factors	KLF2 → antiinflammatory genes↑ NRF2 → antioxidative genes↑	AP-1 → cell differentiation, proliferation and apoptosis↑ NF-ᴋB → proinflammatory genes↑	
Cellular responses	EC permeability↓ Growth factor production↑ Mechanoactivation of eNOS → NO production↑ Arterial remodeling↑ Timp3 expression↑ → inhibition of MMPs Platelet activation↑	EC permeability↑ EC/SMC proliferation↑ Downregulation of eNOS → NO production↓ ROS production↑ Mechanoactivation of NADPH oxidase → LDL oxidation↑ Stimulation of oxLDL uptake	
Associated plaque characteristics	Endothelial denudation Hemorrhage and calcification Fibrous cap thinning Thrombus formation	Endothelial dysfunction Intimal thickening Collagen deposition Plaque inflammation	

EC: endothelial cell, eGCX: endothelial glycocalyx, eNOS: endothelial nitric oxide synthase, KLF2: Krüppel-like factor 2, MMP: matrix metalloprotease, NADPH: nicotinamide adenine dinucleotide phosphate, NO: nitric oxide, NRF2: nuclear factor erythroid 2-related factor 2, oxLDL: oxidized low-density lipoprotein, Re: Reynolds number, ROS: reactive oxygen species, SMC: smooth muscle cell, +: is observed, −: is not observed, ↑: upregulation, ↓: downregulation.
